# Alterations in Cortical Thickness in Young Male Patients With Childhood-Onset Adult Growth Hormone Deficiency: A Morphometric MRI Study

**DOI:** 10.3389/fnins.2019.01134

**Published:** 2019-10-22

**Authors:** Hongbo Yang, Kang Li, Xinyu Liang, Bin Gu, Linjie Wang, Gaolang Gong, Feng Feng, Hui You, Bo Hou, Fengying Gong, Huijuan Zhu, Hui Pan

**Affiliations:** ^1^Department of Endocrinology, Key Laboratory of Endocrinology of National Health Commission, The Translational Medicine Center of PUMCH, Peking Union Medical College Hospital, Chinese Academy of Medical Sciences & Peking Union Medical College, Beijing, China; ^2^State Key Laboratory of Cognitive Neuroscience and Learning & IDG/McGovern Institute for Brain Research, Beijing Normal University, Beijing, China; ^3^Department of Radiology, Peking Union Medical College Hospital, Chinese Academy of Medical Sciences & Peking Union Medical College, Beijing, China

**Keywords:** childhood-onset adult growth hormone deficiency, cortical thickness, structure MRI, growth hormone, insulin-like growth factor 1

## Abstract

**Background:**

The growth hormone (GH)/insulin-like growth factor-1 (IGF-1) axis plays an important role in brain structure and maintenance of brain function. There is a close correlation between serum GH and IGF1 levels and age-related cognitive function. The effects of childhood-onset growth hormone deficiency (GHD)on brain morphology are underestimated so far.

**Methods:**

In this cross-sectional study, T1-weighted magnetic resonance imaging was assessed in 17 adult males with childhood-onset GHD and 17 age and gender-matched healthy controls. The cortical thickness was analyzed and compared between the two groups of subjects. Effects of disease status and hormone levels on cortical thickness were also evaluated.

**Results:**

Although there was no difference in whole brain volume or gray matter volume between the two groups, there was decreased cortical thickness in the parahippocampal gyrus, posterior cingulate gyrus and occipital visual syncortex in the adult growth hormone deficiency (AGHD) group, and increased cortical thickness in a partial area of the frontal lobe, parietal lobe and occipital visual syncortex in AGHD group. Cortical thickness of the posterior cingulum gyrus was prominently associated with FT3 serum levels only in control group after adjusting of IGF-1 levels.

**Conclusion:**

These results suggest that young adult male patients with childhood-onset GHD have alterations in cortical thickness in different brain lobes/regions.

## Introduction

Adult growth hormone deficiency (AGHD) is a debilitating condition that occurs due to insufficient secretion of GH from the pituitary gland. AGHD is caused by hereditary defects of GH synthesis and secretion or resulted from tumor, head injury, and head radiation or pituitary surgery. Besides metabolic disorders, osteoporosis, sarcopenia and decreased quality of life, impaired cognitive function is one of the important characteristics of AGHD (Molitch et al., 2011; Uzunova et al., 2015). AGHD can be divided into childhood-onset and adulthood-onset, according to the age of onset. Most cases of isolated growth hormone deficiency (GHD) in childhood are idiopathic and transient, while in patients with multiple pituitary hormone deficiency (MPHD), GHD generally persists into adulthood (Berberoğlu et al., 2008; Yang et al., 2017).

Brain morphology and function could be influenced during early stages of development in the uterus and can dynamically change during adulthood (Buchanan et al., 1992). GH is a peptide synthesized by the anterior pituitary that stimulates insulin-like growth factor-1 (IGF-1) secretion from the liver. GH receptor and IGF1 receptor have been localized in brain regions important in cognitive functioning, including the hippocampus, amygdala and parahippocampal areas (Adem et al., 1989). The GH/IGF1-axis plays an essential role during early brain development and persists in several brain regions with continuous renewal and remodeling during adulthood (Wrigley et al., 2017). In neural stem cells, IGF-1 is a controlling switch for long-term proliferation (Supeno et al., 2013). A higher serum IGF-1 level is associated with better working memory and mental processing speed in healthy subjects (Aleman et al., 1999). Morphologically, higher serum IGF-1 levels are associated with significantly increased cerebral blood flow in the left dorsolateral prefrontal cortex and left premotor cortex (Arwert et al., 2005).

Despite the role of the GH/IGF-1 axis in normal brain development and function, the effects of GHD on brain structure are not fully understood thus far. It was reported that reduced thalamic and globus pallidum volumes were related to deficits in cognitive function and motor performance in children with isolated GHD (Webb et al., 2012). In patients with MPHD, GHD is usually permanent and recombinant human GH (rhGH) replacement is usually stopped after completion of linear growth. Higher incidence of mental disorders and increased prevalence of cognitive dysfunction were reported in hypopituitary women with GH deficiency (Bülow et al., 2002). There were also several studies to evaluate the effects of GH replacement therapy in cognitive function improvement in AGHD patients. But interpretation of data is complicated by participants selection as well as neuropsychological tests used in these studies (van Dam, 2005). Morphological variations in the brains of patients with childhood-onset AGHD after cessation of rhGH treatment have received little research attention to date.

Recently, voxel-based statistical analysis based on an ^18^F-FDG PET study found that adult patients with GHD due to traumatic brain injury had decreased cerebral glucose metabolism in cortical areas involved in intellectual function, executive function and working memory (Park et al., 2016). No direct assessment of cortical thickness of AGHD patients has been reported so far. Cortical thickness provides direct and reliable information about the density, size and arrangement of cortical cells, thus yielding insights into the regional integrity of the cerebral cortex. One of the main challenges in evaluation of brain structure variations in patients with AGHD is the lack of homogeneity in etiology, course of disease and variation of hormone replacement therapy. In this cross-sectional study, cortical thickness of adult males with childhood-onset AGHD due to pituitary hypoplasia or pituitary stalk interruption was compared to age- and gender-matched healthy controls. The effects of disease status and hormone levels on cortical thickness were also evaluated.

## Subjects and Methods

### Participants

In this cross-sectional study, a total of 17 consecutive male patients with childhood-onset AGHD were enrolled from January 2011 to March 2013 in Peking Union Medical College Hospital (Wilson, 1993). Inclusion criteria included: (1) all patients fulfilled the diagnosis criteria of AGHD according to the clinical guideline of the American Endocrine Society (Molitch et al., 2011). Twelve patients underwent insulin tolerance test and peak value of GH was lower than 3 ng/ml. The diagnosis was confirmed in the other five patients with IGF-1 levels below the age-adjusted normal range and deficiencies in three or more pituitary axes at the same time., (2) linear growth was finished and bone age ≥18 years, (3) rhGH had been stopped at least one year after final height, (4) prednisone, L-thyroxine and testosterone replacement therapy were sustained as needed at least one year before enrollment, and (5) right handedness and with no contraindication to MRI. A total of 17 healthy age-matched male subjects were enrolled as controls. All controls were right-handed and education-matched. Medical history of major neurological or psychiatric disorders was documented in neither the AGHD group nor the control group. The study protocol was approved by the Ethics Committees of Peking Union Medical College Hospital. Written informed consent was obtained from all subjects and all data were de-identified before analysis.

### Clinical Assessment

Main demographic data were obtained from medical charts. Weight and height were measured in the early morning. Body mass index (BMI) was calculated as weight (kg) divided by height (m) squared. Questionnaires about education levels were assessed. Blood samples were also obtained in early morning. Serum levels of IGF1, free thyroxine (FT4) free triiodothyronine (FT3), thyrotropin (TSH), total testosterone and fasting blood glucose were tested in the department of the clinical laboratory using standard protocols (Zhu et al., 2017).

### MRI Image Acquisition

Imaging was performed on a 3 T MR scanner (General Electric Medical Systems, GE sign VH/I Excite I 3.0 T). Lying in supine position, participants fixed their heads using foam padding to avoid head movement. A scout for anterior commissure-posterior alignment was done first, then sagittal three-dimensional (3D) volumetric T1-weighted magnetization-prepared rapid acquisition gradient echo (MPRAGE) images were obtained (128 sagittal slices, repetition time (TR) = 2530 ms, echo time (TE) = 3.39 ms, inversion time (TI) = 1100 ms, slice thickness = 1.33 mm, field of view (FOV) = 256 × 256 mm^2^, voxel size = 1.33 × 1.00 × 1.00 mm^3^).

### Image Processing

Cortical thickness was extracted from the structural MR T1 images by using a routine pipeline of the CIVET software (version 1.1.9; Montreal Neurological Institute at McGill University, Canada). A flowchart for cortical thickness assessment was described previously (Liu et al., 2014). In brief, the original images were linear registered to stereotaxic space using the average NMI-ICBM152 model, for obtaining better segmentation following (Collins et al., 1994). Then the non-uniformity artifacts of images were corrected by the Non-parametric Non-uniform intensity Normalization (N3) algorithm (Sled et al., 1998), which was shown to be accurate and robust (Sled et al., 1998). By using an advanced neural classifier, the registered and corrected images were then segmented into white matter (WM), grey matter (GM), cerebrospinal fluid (CSF) and background (Zijdenbos et al., 2002; Jussi et al., 2004). The inner and outer GM surfaces were extracted from each hemisphere by using the constrained Laplacian-based automated segmentation with proximities (CLASP) algorithm (MacDonald et al., 2000; Kim et al., 2005). Specifically, the inner surface was reconstructed by deforming a spherical polygon model to the WM/GM boundary with a total of 81,924 vertices (40,962 of each hemisphere). The outer surface was then initiated from the inner surface and was expanded to the GM/CSF fluid boundary along the Laplacian field generated from a skeletonized CSF fraction image (Cherng-Min and Shu-Yen, 2001; Cherng-Min et al., 2002). Cortical thickness was defined as the Euclidean distance between linked vertices on the inner and outer surfaces (Lerch and Evans, 2005). For each subject, we visually evaluated the results of segmentation and reconstruction using the recommended method, and ensured that no error and failure was appeared. Finally, a 20-mm 2-D smoothing was applied on cortical thickness map for less noise and better sensitivity in statistics analysis.

### Statistical Analysis

Numerical data are expressed as the mean ± standard deviation, and *t*-tests were used to compare data between two groups. A general linear model (GLM) was used for thickness modeling at each surface vertex as a linear combination of effects related to variables of interests and effects of potential confounds (years of education, age and BMI). The main effect of groups and the interaction between groups and hormone levels were tested. As described previously, a random field theory (RFT)-based method was used at the cluster level for all cortical analysis in order to correct for multiple comparisons. Cortical clusters with an FWE-corrected *p* < 0.05 were considered significant. After the interaction analyses, we performed the *post hoc* tests for the significant clusters. We used the Pearson’s correlations (r) to quantify the relationship between hormone levels and averaged cortical thickness of clusters within each group. All statistical analyses were carried out using the SurfStat toolbox^1^ in MATLAB (Yaojing et al., 2015).

## Results

### Demographic, Clinical and Biochemical Characteristics

Demographic and clinical characteristics are listed in Table 1. Nine of AGHD patients (9/17) were born with breech or foot presentation. MRI revealed pituitary hypoplasia or pituitary stalk interruption in all these patients. All patients had MPHD. All patients had MPHD and sustained glucocorticoid and levothyroxine replacement since childhood. Testosterone replacement was started at 18 years old and after completion of linear growth in all patients. AGHD group had received 32.5 ± 3.5 months of replacement therapy with rhGH and stopped after completion of linear growth.

**TABLE 1:** General baseline data and distribution of all subjects.

	**AGHD group (*n* = 17)**	**Controls (*n* = 17)**	***p***
Age (years)	25.0 ± 4.6	24.0 ± 3.9	0.87
Height (cm)	163.5 ± 6.7	173.0 ± 5.1	<0.001
Weight (kg)	62.1 ± 10.3	71.5 ± 8.2	0.006
BMI (kg/m^2^)	23.2 ± 3.3	23.9 ± 2.8	0.47
Education duration (years)	11.4 ± 3.2	12.1 ± 3.0	0.55
Fasting blood glucose (mmol/L)	5.0 ± 0.5	5.1 ± 0.4	0.65
IGF-1 (ng/ml)	49.1 ± 26.1	234.0 ± 88.1	<0.001
IGF-1 SDS	−5.04 ± 1.60	−0.35 ± 1.17	<0.001
FT3 (pg/ml)	2.8 ± 0.7	3.7 ± 0.3	<0.001
FT4 (ng/dl)	0.9 ± 0.4	1.0 ± 0.2	<0.001
TSH (μIU/ml)	1.4 ± 1.2	1.9 ± 0.8	0.18
Testosterone (ng/dl)	462.9 ± 161.3	478.0 ± 121.4	0.75

There was no difference in chronological age (25.0 ± 3.6 vs. 24.0 ± 3.9 years, *p* = 0.87) or school-education years between AGHD group and the control group. The AGHD group had a 9.5 cm lag in final height (163.5 ± 6.7 vs. 173.0 ± 5.1 cm, *p* < 0.001) and lower body weight (62.1 ± 10.3 vs. 71.5 ± 8.2 kg, *p* = 0.006) compared with the control group. BMI was similar between the two groups. AGHD group presented with a significant decrease in IGF1 levels at the time of the study (49.1 ± 26.1 vs. 234.0 ± 88.1 ng/ml, *p* < 0.001). With monthly intramuscular injections of testosterone undecanoate, serum levels of the AGHD group were similar to that of the control group (*p* = 0.75). With daily replacement with levothyroxine, serum levels of FT4 and FT3 in the AGHD group were in the lower quartile of the normal range, but were significantly lower than controls (*p* < 0.001).

### Alterations of Brain Volumes

As shown in Table 2, there was no difference in whole brain volume (*p* = 0.20) or gray matter volume (*p* = 0.08) between the AGHD and control groups.

**TABLE 2:** Comparison of whole brain volume and gray matter between study groups.

	**AGHD group (*n* = 17)**	**Control group (*n* = 17)**	***p***
Whole brain volume (mm^3^)	1526.54 ± 134.38	1464.61 ± 141.50	0.20
Gray matter volume (mm^3^)	697.69 ± 59.09	661.08 ± 59.09	0.08

### Alteration of Cortical Thickness in Different Lobes and Regions

**FIGURE 1:**
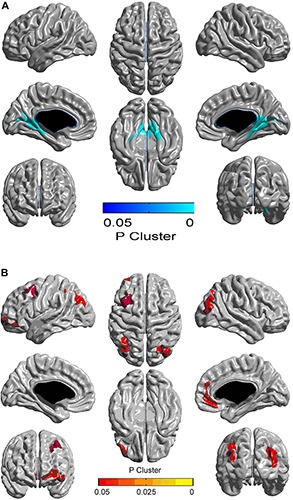
Alterations in cortical thickness in young male patients with childhood-onset adult growth hormone deficiency. Clusters with FWE-corrected *p* < 0.05 are shown, and the color corresponds to the *p*-value. Areas in blue represent decreased cortical thickness in AGHD compared to controls, and yellow area represent increased thickness. **(A)** Clusters of significantly reduced cortical thickness in the AGHD group compared to controls. **(B)** Clusters of significantly increased cortical thickness in the AGHD group compared to controls.

Compared with the control group, the AGHD group showed decreased cortical thickness in the parahippocampal gyrus (Brodmann’s area 27), posterior cingulate gyrus (Brodmann’s area 29, 30) and occipital visual syncortex Brodmann’s area 17, 18, 19)(Figure 1A and Table 3). While in the partial area of the frontal lobe (Brodmann’s area 9, 10, 11, 47), parietal lobe (Brodmann’s area 7,39) and occipital visual syncortex (Brodmann’s area 17, 18, 19), the AGHD group showed increased cortical thickness (Figure 1B and Table 3). The significant clusters are different and exclusive, although some of them belong to same Brodmann’s region like Brodmann’s area 17, 18, 19. There was no overlap between these parts.

**TABLE 3:** Regional variation of cerebral cortex thickness in AGHD patients compared with normal control.

**Cortex thickness: AGHD vs. control**	**Brodmann’s areas**	**Cluster size**	**Peak *x***	**Peak *y***	**Peak *z***	**Peak *T*-value**
AGHD > control	7, 10, 11, 19, 39	1225	−37	−57	44	4.63
	17, 18, 19	731	45	−79	−18	5.13
	9	191	−27	14	49	3.8
	47	157	−46	44	−16	4.59
AGHD < control	17, 18, 19, 27, 29, 30	2720	21	−55	1	7.23
	23	115	−21	−69	5	4.89

### Interaction Analysis

**FIGURE 2:**
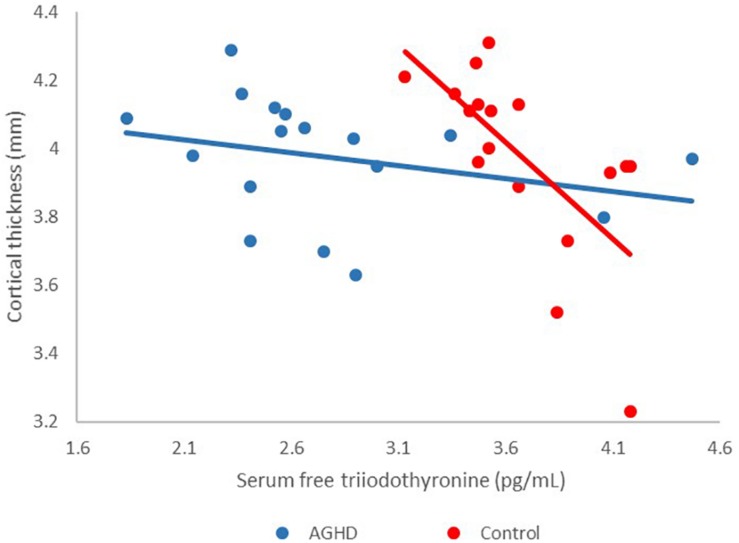
Effects of interaction between groups and serum FT3 levels on cortical thickness. Red points, controls; blue points, AGHD patients.

The associations between cortical thickness and serum IGF-1 levels were not significantly different between the two groups after adjusting for serum levels of FT3. Meanwhile associations between groups and serum FT3 levels and the effects on cortical thickness was found in our data (*F* = 16.55, *df* = 1, 29, *p* < 0.001). Cortical thickness of the posterior cingulum gyrus was prominently only associated with serum levels of FT3 in control group after adjusting for IGF-1 levels (AGHD group, *r* = −0.28, *p* = 0.27; control group, *r* = −0.66, *p* = 0.004; Figure 2).

## Discussion

This cross-sectional study focused on cortical thickness alterations of young male patients with childhood-onset AGHD after cessation of rhGH replacement therapy. Our results showed that: (1) Compared to age-, gender- and education-matched controls, the AGHD group had significantly decreased IGF1 levels. With routine replacement of levothyroxine, serum FT4 and FT3 levels in the AGHD group were in the lower quartile of the normal range, but were significantly lower than for the controls. (2) Although there was no difference in whole brain volume or gray matter volume between the two groups, there was a decreased cortical thickness in the parahippocampal gyrus, posterior cingulate gyrus and occipital visual syncortex in the AGHD group, as well as an increased cortical thickness in the partial area of the frontal lobe, parietal lobe and occipital visual syncortex. (3) Cortical thickness of the posterior cingulum gyrus was prominently associated with serum levels of FT3 in the control group after adjusting of IGF-1 levels, and the relationship was not observed in AGHD group.

The GH/IGF-1 axis plays an important role in brain development and maintenance of cognitive function (Annenkov, 2009). The GH receptor is prominently expressed in the hypothalamus, hippocampus, putamen and choroid plexus (Lai et al., 1993). Meanwhile, IGF-1 receptors are expressed in cerebellum, prefrontal cortex, caudate nucleus, amygdala, hippocampal and parahippocampal area (Bondy and Lee, 1993). During adolescence, gene expression in hippocampal area was reported to be regulated by IGF1 (Yan et al., 2011). In mice, the GH/IGF-1 axis plays a specific role in corticospinal tract development, and corticospinal axon growth will be impaired as the result of an interruption to IGF-1 signaling (Ozdinler and Macklis, 2006). However, how the disease process impacts the structural integrity of the brain in humans still needs further investigation. In children with isolated GHD, it was reported that there were decreased volumes in the right hippocampus, right pallidum and left thalamus, compared to controls (Webb et al., 2012). However, no previous studies have evaluated the structural characteristics in patients with MPHD after cessation of rhGH replacement therapy so far. In our study, all subjects had childhood-onset MPDH and stopped rhGH replacement therapy after completion of linear growth. Cortical thickness in the parahippocampal gyrus, posterior cingulate gyrus and occipital visual syncortex decreased, while cortical thickness in partial area of the frontal lobe, parietal lobe, occipital visual syncortex and angular gyrus increased in our AGHD subjects. The variations of cortical thickness in the cingulate cortex and frontal lobe are consistent with previous reports from young patients with GH receptor deficiency (Nashiro et al., 2017), since IGF-1 signaling is supposed to play an important role in brain regeneration in these areas. However, variations of cortical thickness in the occipital visual syncortex, angular gyrus and parahippocampal gyrus have not been described in AGHD patients before, and functional implications need to be further investigated.

Patients with AGHD were reported to have impaired cognitive function (memory and attention) in several neuropsychological studies, and there are several reports evaluating GH replacement therapy in cognitive function improvement in AGHD patients (Falleti et al., 2006). Several structures are known to play important roles in cognitive function, including memory and learning, and distribution of IGF-1 receptors reportedly interact with the hippocampal cholinergic system and are involved in cognitive development (Araujo et al., 1989; Bondy and Lee, 1993). In the elderly, higher concentrations of serum IGF-1 levels were associated with better performance on cognitive function tests, which suggested that the GH/IGF-1 axis may affect cognitive function throughout life (Al-Delaimy et al., 2009). In our AGHD subjects, decreased cortical thickness was found in parahippocampal gyrus, posterior cingulate gyrus and occipital visual syncortex. The parahippocampal gyrus surrounds the hippocampus and is part of the limbic system and plays an important role in memory encoding and retrieval. It was reported that parahippocampal cortical thickness was reduced in people at ultra-high risk of psychosis (Tognin et al., 2014). The posterior cingulate cortex is made up of an area around the midline of the brain, communicates with various brain networks simultaneously and is involved in diverse functions (Leech et al., 2012). Reduced cortical thickness of the posterior cingulate was reported in patients with improved migraines, and thus, it was hypothesized to be related with chronic pain development (Amaral et al., 2018). The occipital visual syncortex is located in the occipital lobe and is involved in visual information processing. Childhood onset of blindness significantly affects the cortical thickness of the primary visual cortex (Li et al., 2017). Cortical thickness changes of the above three areas have not been evaluated in GHD thus far. There are also some regions with increased cortical thickness, some of which play different roles in cognition, semantic processing and sensory afferents. The significance of these alterations, and whether rhGH replacement has any effect on them, needs further assessments.

At the same time, the associations between cortical thickness and serum IGF-1 levels were not significantly different between two groups after adjusting for serum levels of FT3. Meanwhile an association between groups and serum FT3 levels and the effects on cortical thickness was found in our data. Cortical thickness of posterior cingulum gyrus was prominently associated with serum levels of FT3 only in the control group after adjusting for IGF-1 levels. These results may suggest that the effects of the thyroid hormone on cortical thickness in AGHD patients have been lessened in the presence of GHD. The thyroid hormone plays an essential role in early stages of brain development (Cuevas et al., 2005), and structures including the hippocampus, striatum and cortex are reported to be abnormal in rats (Rami et al., 1986), mice (Gil-Ibañez et al., 2013) and children (Clairman et al., 2015) with hypothyroidism. Children with congenital hypothyroidism showed cortical thinning or thickening in a few areas (Clairman et al., 2015), which are different from all the changed areas found in our study. The interaction between the GH and thyroid hormone and its effects on the maintenance of cortical thickness in different regions of the brain is not clear to date, and animal models may provide further information for this interplay.

There are several limitations in this study. The first and most important one is the limited number of subjects, however, there was relatively good homogeneity in the etiology, age of onset, duration of rhGH treatment and time course since cessation of rhGH treatment, which provided a general uniform background for morphology evaluation. Stable rules in interaction analysis will be warranted with more samples in further investigation. Another limitation is the cross-sectional nature of this study, which does not provide a cause-effect rationale for GHD and changes in cortical thickness. The third limitation is cognitive tests had not been assessed in AGHD patients since alterations of cortical thickness may be related to changed cognitive function. Further prospective studies are needed to shed new lights on the effects of GH replacement therapy on brain structure changes and cognitive function improvement in AGHD patients.

In summary, our findings indicate that young male patients with childhood-onset AGHD who are no longer receiving GH replacement have alterations in cortical thickness in different brain lobes/regions. It will be important to expand on these results in a larger sample size, ideally incorporating not only structural, but also functional brain measures.

## Data Availability Statement

The raw data supporting the conclusions of this manuscript will be made available by the authors, without undue reservation, to any qualified researcher.

## Ethics Statement

The study protocol was approved by the Ethics Committees of Peking Union Medical College Hospital. Written informed consent was obtained from all subjects and all data were de-identified before analysis.

## Author Contributions

HY and KL collected the clinical information, image data, and drafted the manuscript. XL, BG, and GG analyzed the MRI image data. LW and FG helped with clinical and biochemical data analysis. FF, HY, and BH were responsible for the MRI image acquisition. HZ and HP designed the study protocol and revised the manuscript.

## Conflict of Interest

The authors declare that the research was conducted in the absence of any commercial or financial relationships that could be construed as a potential conflict of interest.

## Footnotes

1 http://www.math.mcgill.ca/keith/surfstat/

## References

[B1] Adem A. Jossan S. S. d’Argy R. Gillberg P. G. Nordberg A. Winblad B. (1989). Insulin-like growth factor 1 (IGF-1) receptors in the human brain: quantitative autoradiographic localization. *Brain Res.* 503 299–303. 10.1016/0006-8993(89)91678-8 2557967

[B2] Al-Delaimy W. K. von Muhlen D. Barrett-Connor E. (2009). Insulinlike growth factor-1, insulinlike growth factor binding protein-1, and cognitive function in older men and women. *J. Am. Geriatr. Soc.* 57 1441–1446. 10.1111/j.1532-5415.2009.02343.x 19515112 PMC2728156

[B3] Aleman A. Verhaar H. J. De Haan E. H. De Vries W. R. Samson M. M. Drent M. L. (1999). Insulin-like growth factor-I and cognitive function in healthy older men. *J. Clin. Endocrinol. Metab.* 84 471–475. 10.1210/jc.84.2.471 10022403

[B4] Amaral V. C. G. Tukamoto G. Kubo T. Luiz R. R. Gasparetto E. Vincent M. B. (2018). Migraine improvement correlates with posterior cingulate cortical thickness reduction. *Arq. Neuropsiquiatr.* 76 1501–1557. 10.1590/0004-282x20180004 29809228

[B5] Annenkov A. (2009). The Insulin-Like Growth Factor (IGF) Receptor Type 1 (IGF1R) as an Essential Component of the Signalling Network Regulating Neurogenesis. *Mol. Neurobiol.* 40 195–215. 10.1007/s12035-009-8081-0 19714501

[B6] Araujo D. M. Lapchak P. A. Collier B. Chabot J. G. Quirion R. (1989). Insulin-like growth factor-1 (somatomedin-C) receptors in the rat brain: distribution and interaction with the hippocampal cholinergic system. *Brain Res.* 484 130–138. 10.1016/0006-8993(89)90355-7 2540883

[B7] Arwert L. I. Veltman D. J. Deijen J. B. Lammertsma A. A. Jonker C. Drent M. L. (2005). Memory performance and the growth hormone/insulin-like growth factor axis in elderly: a positron emission tomography study. *Neuroendocrinology* 81 31–40. 10.1159/000084872 15809510

[B8] Berberoğlu M. Sıklar Z. Darendeliler F. Poyrazoğlu S. Darcan S. Işgüven P. (2008). Evaluation of permanent growth hormone deficiency (GHD) in young adults with childhood onset GHD: a multicenter study. *J. Clin. Res. Pediatr. Endocrinol.* 1 30–37. 10.4008/jcrpe.v1i1.7 21318062 PMC3005632

[B9] Bondy C. A. Lee W. H. (1993). Patterns of insulin-like growth factor and igf receptor gene expression in the brain: functional implications. *Ann. N. Y. Acad. Sci.* 692 33–43. 10.1111/j.1749-6632.1993.tb26203.x 8215043

[B10] Buchanan C. M. Eccles J. S. Becker J. B. (1992). Are adolescents the victims of raging hormones: evidence for activational effects of hormones on moods and behavior at adolescence. *Psychol. Bull.* 111 62–107. 10.1037//0033-2909.111.1.62 1539089

[B11] Bülow B. Hagmar L. Ørbaek P. Osterberg K. Erfurth E. M. (2002). High incidence of mental disorders, reduced mental well-being and cognitive function in hypopituitary women with GH deficiency treated for pituitary disease. *Clin. Endocrinol.* 56 183–193. 10.1046/j.0300-0664.2001.01461.x 11874409

[B12] Cherng-Min M. Shu-Yen W. (2001). A medial-surface oriented 3-d two-subfield thinning algorithm. *Pattern Recognit. Lett.* 22 1439–1446. 10.1016/s0167-8655(01)00083-6

[B13] Cherng-Min M. Shu-Yen W. Jiann-Der L. (2002). Three-dimensional topology preserving reduction on the 4-subfields. *IEEE Trans. Pattern* 24 1594–1605. 10.1109/tpami.2002.1114851

[B14] Clairman H. Skocic J. Lischinsky J. E. Rovet J. (2015). Do children with congenital hypothyroidism exhibit abnormal cortical morphology. *Pediatr. Res.* 78 286–297. 10.1038/pr.2015.93 25978801

[B15] Collins D. L. Neelin P. Peters T. M. Evans A. C. (1994). Automatic 3D intersubject registration of MR volumetric data in standardized talairach space. *J. Comput. Assist. Tomogr.* 18 192–205. 10.1097/00004728-199403000-00005 8126267

[B16] Cuevas E. Ausó E. Telefont M. Morreale de Escobar G. Sotelo C. Berbel P. (2005). Transient maternal hypothyroxinemia at onset of corticogenesis alters tangential migration of medial ganglionic eminence-derived neurons. *Eur. J. Neurosci.* 22 541–551. 10.1111/j.1460-9568.2005.04243.x 16101736

[B17] Falleti M. G. Maruff P. Burman P. Harris A. (2006). The effects of growth hormone (GH) deficiency and GH replacement on cognitive performance in adults: a meta-analysis of the current literature. *Psychoneuroendocrinology* 31 681–691. 10.1016/j.psyneuen.2006.01.005 16621325

[B18] Gil-Ibañez P. Morte B. Bernal J. (2013). Role of Thyroid hormone receptor subtypes α and β on gene expression in the cerebral cortex and striatum of postnatal mice. *Endocrinology* 154 1940–1947. 10.1210/en.2012-2189 23493375

[B19] Jussi T. Alex Z. Alan E. (2004). Fast and robust parameter estimation for statistical partial volume models in brain MRI. *Neuroimage* 23 84–97. 10.1016/j.neuroimage.2004.05.007 15325355

[B20] Kim J. S. Singh V. Lee J. K. Lerch J. Ad-Dab’bagh Y. MacDonald D. (2005). Automated 3-D extraction and evaluation of the inner and outer cortical surfaces using a Lapcalcian map and partial volume effect classification. *Neuroimage* 27 210–221. 10.1016/j.neuroimage.2005.03.036 15896981

[B21] Lai Z. Roos P. Zhai O. Olsson Y. Fhölenhag K. Larsson C. (1993). Age-related reduction of human growth hormone-binding sites in the human brain. *Brain Res.* 621 260–266. 10.1016/0006-8993(93)90114-3 8242339

[B22] Leech R. Braga R. Sharp D. J. (2012). Echoes of the brain within the posterior cingulate cortex. *J. Neurosci.* 32 215–222. 10.1523/JNEUROSCI.3689-11.2012 22219283 PMC6621313

[B23] Lerch J. P. Evans A. C. (2005). Cortical thickness analysis examined through power analysis and a population simulation. *Neuroimage* 24 163–173. 10.1016/j.neuroimage.2004.07.045 15588607

[B24] Li Q. Song M. Xu J. Qin W. Yu C. Jiang T. (2017). Cortical thickness development of human primary visual cortex related tothe age of blindness onset. *Brain Imaging Behav.* 11 1029–1036. 10.1007/s11682-016-9576-8 27468855

[B25] Liu Y. Xie T. He Y. Duan Y. Huang J. Ren Z. (2014). Cortical Thinning Correlates with Cognitive Change in Multiple Sclerosis but not in Neuromyelitis Optica. *Eur. Radiol.* 24 2334–2343. 10.1007/s00330-014-3239-1 24906701

[B26] MacDonald D. Kabani N. Avis D. Evans A. C. (2000). Automated 3-D extraction of inner and outer surfaces of cerevral cortex from MRI. *Neuroimage* 12 340–356. 10.1006/nimg.1999.0534 10944416

[B27] Molitch M. E. Clemmons D. R. Malozowski S. Merriam G. R. Shalet S. M. Vance M. L. (2011). Evaluation and treatment of adult growth hormone deficiency: an Endocrine Society clinical practice guideline. *J. Clin. Endocrinol. Metab.* 96 1587–1609. 10.1210/jc.2011-0179 21602453

[B28] Nashiro K. Guevara-Aguirre J. Braskie M. N. Hafzalla G. W. Velasco R. Balasubramanian P. (2017). Brain structure and function associated with younger adults in growth hormone receptor-deficient humans. *J. Neurosci.* 37 1696–1707. 10.1523/JNEUROSCI.1929-16.2016 28073935 PMC5320603

[B29] Ozdinler P. H. Macklis J. D. (2006). IGF-I specifically enhances axon outgrowth of corticospinal motor neurons. *Nat. Neurosci.* 9 1371–1381. 10.1038/nn1789 17057708

[B30] Park K. D. Lim O. K. Yoo C. J. Kim Y. W. Lee S. Park Y. (2016). Voxel-based statistical analysis of brain metabolism in patients with growth hormone deficiency after traumatic brain injury. *Brain Inj.* 30 407–413. 10.3109/02699052.2015.1127997 26910852

[B31] Rami A. Patel A. J. Rabié A. (1986). Thyroid hormone and development of the rat hippocampus: morphological alterations in granule and pyramidal cells. *Neuroscience* 19 1217–1226. 10.1016/0306-4522(86)90135-1 3822116

[B32] Sled J. G. Zijdenbos A. P. Evans A. C. (1998). A nonparametric method for automatic correction of intensity nonuniformity in MRI data. *IEEE Trans. Med. Imaging* 17 87–97. 10.1109/42.668698 9617910

[B33] Supeno N. E. Pati S. Hadi R. A. Ghani A. R. Mustafa Z. Abdullah J. M. (2013). IGF-1 acts as controlling switch for long-term proliferation and maintenance of EGF/FGF-responsive striatal neural stem cells. *Int. J. Med. Sci.* 10 522–531. 10.7150/ijms.5325 23532711 PMC3607237

[B34] Tognin S. Riecher-Rössler A. Meisenzahl E. M. Wood S. J. Hutton C. Borgwardt S. J. (2014). Reduced parahippocampal cortical thickness in subjects at ultra-high risk for psychosis. *Psychol. Med.* 44 489–498. 10.1017/S0033291713000998 23659473 PMC3880065

[B35] Uzunova I. Kirilov G. Zacharieva S. Shinkov A. Borissova A. M. Kalinov K. (2015). Individual risk factors of the metabolic syndrome in adult patients with growth hormone deficiency – a cross-sectional case-control study. *Exp. Clin. Endocrinol. Diabetes* 123 39–43. 10.1055/s-0034-1390460 25412168

[B36] van Dam P. S. (2005). Neurocognitive function in adults with growth hormone deficiency. *Horm. Res.* 64(Suppl. 3), 109–114. 10.1159/000089326 16439853

[B37] Webb E. A. O’Reilly M. A. Clayden J. D. Seunarine K. K. Chong W. K. Dale N. (2012). Effect of growth hormone deficiency on brain structure, motor function and cognition. *Brain* 135 216–227. 10.1093/brain/awr305 22120144

[B38] Wilson J. D. (1993). Peking Union Medical College Hospital, a palace of endocrine treasures. *J. Clin. Endocrinol. Metab.* 76 815–816. 10.1210/jcem.76.4.8473387 8473387

[B39] Wrigley S. Arafa D. Tropea D. (2017). Insulin-like growth factor 1: at the crossroads of brain development and aging. *Front. Cell Neurosci.* 11:14. 10.3389/fncel.2017.00014 28203146 PMC5285390

[B40] Yan H. Mitschelen M. Bixler G. V. Brucklacher R. M. Farley J. A. Han S. (2011). Circulating IGF1 regulates hippocampal IGF1 levels and brain gene expression during adolescence. *J. Endocrinol.* 211 27–37. 10.1530/JOE-11-0200 21750148 PMC3395434

[B41] Yang H. Zhu H. Yan K. Pan H. (2017). Childhood-onset adult growth hormone deficiency: clinical, hormonal, and radiological assessment in a single center in China. *Horm. Res. Paediatr.* 88 155–159. 10.1159/000478527 28719905

[B42] Yaojing C. Peng L. Bin G. Zhen L. Xin L. Alan C. (2015). The effects of an APOE promoter polymorphism on human cortical morphology during nondemented aging. *J. Neurosci.* 35 1423–1431. 10.1523/JNEUROSCI.1946-14.2015 25632120 PMC6795261

[B43] Zhu H. Xu Y. Gong F. Shan G. Yang H. Xu K. (2017). Reference ranges for serum insulin-like growth factor 1 (IGF-1) in healthy Chinese adults. *PLoS One* 12:e0185561. 10.1371/journal.pone.0185561 28976993 PMC5627923

[B44] Zijdenbos A. P. Forghani R. Evans A. C. (2002). Automatic “pipeline” analysis of 3-D MRI data for clinical trials: application to multiple sclerosis. *IEEE Trans. Med. Imaging* 21 1280–1290. 12585710 10.1109/TMI.2002.806283

